# Recent progress in tuberculosis diagnosis: insights into blood-based biomarkers and emerging technologies

**DOI:** 10.3389/fcimb.2025.1567592

**Published:** 2025-05-08

**Authors:** Zewei Yang, Jingjing Li, Jiawen Shen, Huiru Cao, Yuhan Wang, Sensen Hu, Yulu Du, Yange Wang, Zhongyi Yan, Longxiang Xie, Qiming Li, Salwa E. Gomaa, Shejuan Liu, Xianghui Li, Jicheng Li

**Affiliations:** ^1^ School of Basic Medical Sciences, Henan University, Kaifeng, China; ^2^ Department of Microbiology and Immunology, Faculty of Pharmacy, Zagazig University, Zagazig, Egypt; ^3^ School of Nursing and Health, Henan University, Kaifeng, China; ^4^ Institute of Cell Biology, Zhejiang University, Hangzhou, China

**Keywords:** tuberculosis, blood-based biomarkers, diagnostic technologies, artificial intelligence, global health

## Abstract

Tuberculosis (TB) remains a global health challenge, with timely and accurate diagnosis being critical for effective disease management and control. Recent advancements in the field of TB diagnostics have focused on the identification and utilization of blood-based biomarkers, offering a non-invasive, rapid, and scalable approach to disease detection. This review provides a comprehensive overview of the latest progress in blood-based biomarkers for TB, highlighting their potential to revolutionize diagnostic strategies. Furthermore, we explore emerging technologies such as NGS, PET-CT, Xpert and line probe assays, which have enhanced the sensitivity, specificity, and accessibility of biomarker-based diagnostics. The integration of artificial intelligence (AI) and machine learning (ML) in biomarker analysis is also examined, showcasing its potential to improve diagnostic accuracy and predictive capabilities. This review underscores the need for multidisciplinary collaboration and continued innovation to translate these promising technologies into practical, point-of-care solutions. By addressing these challenges, blood-based biomarkers and emerging technologies hold the potential to significantly improve TB diagnosis, ultimately contributing to global efforts to eradicate this devastating disease.

## 1 Introduction

Tuberculosis (TB) is a chronic infectious disease caused by *Mycobacterium tuberculosis* (*Mtb*), primarily leading to pulmonary infection (Miggiano et al., 2020). Other organs such as the brain, gut, and lymph nodes can also be infected, causing extra-pulmonary tuberculosis (Rodriguez-Takeuchi et al., 2019).According to the World Health Organization (WHO), tuberculosis stands as one of the world’s deadliest infectious diseases, with an estimated 10.6 million individuals infected in 2024 (Ghebreyesus, 2024). Moreover, 1.3 million people, including 167,000 individuals living with HIV, lost their lives to TB. About a quarter of the world’s population harbors latent tuberculosis infection (LTBI), and between 5% to 10% of those with LTBI are expected to develop symptoms and advance to active tuberculosis (aTB) (Getahun et al., 2015; Ying et al., 2022).

**Figure 1:**
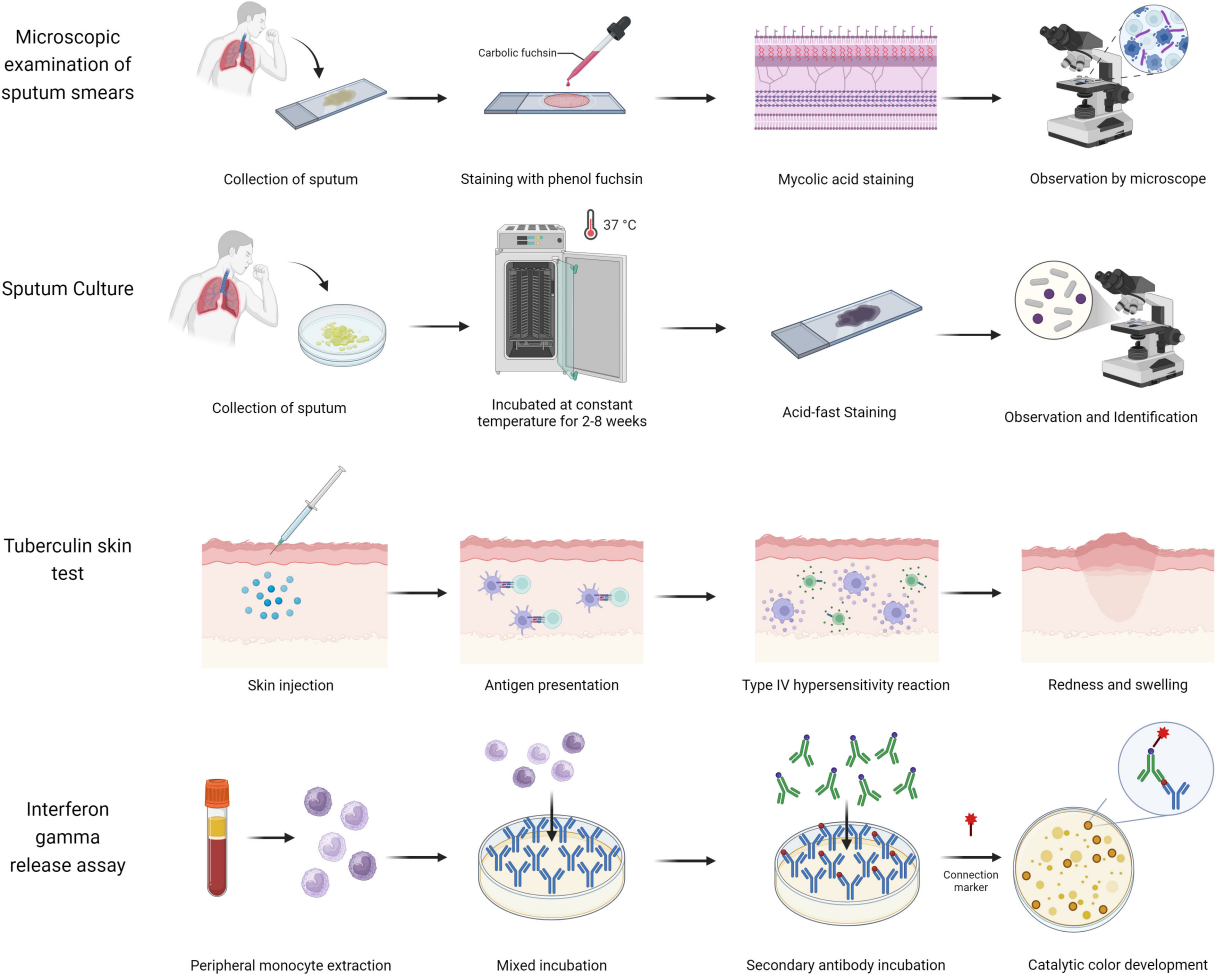
The strategy of existing traditional technologies.

The widespread spread of tuberculosis is mainly due to the lack of a reliable, rapid, and accessible test for diagnosing TB (Nogueira et al., 2022). Traditional diagnostic methods, such as sputum smear microscopy, are commonly used in low- and middle-income countries due to their affordability and simplicity (**Figure 1**, **Supplementary Table S1**). However, these methods have significant limitations, including low sensitivity and a high detection threshold (requiring sputum with more than 1,000 bacilli/mL) (Steingart et al., 2006). Although sputum culture offers high sensitivity (>98%) and a lower detection threshold (>10 bacilli/mL), its lengthy culture duration of 2–8 weeks hinders timely diagnosis and treatment (Domínguez et al., 2023), potentially leading to further TB dissemination. Additionally, tuberculin skin tests (TST) or interferon-gamma release assays (IGRA) are recommended for identifying individuals with LTBI (Hamada et al., 2021). However, TST may yield false-positive results in individuals who have received the BCG vaccine or have been exposed to non-tuberculous mycobacteria (NTM), while IGRA may be less reliable in children under 5 years old and individuals with hematologic disorders or severe immunosuppression (Mandalakas et al., 2011). The emergence of drug-resistant strains of *Mtb* further complicates TB diagnosis and management (Farhat et al., 2024). In 2020, only 71% of patients with confirmed TB were tested for rifampin resistance, with less than half receiving appropriate resistance treatment and a success rate of merely 60% (Bagcchi, 2023). Drug-resistant TB can result in treatment challenges, recurrence of the disease, and the development of extensively drug-resistant tuberculosis (XDR-TB) (Seung et al., 2015), prolonging treatment duration and reducing cure rates (Farhat et al., 2024).

To address these challenges, the current recommendation is to utilize rapid molecular diagnostic tests, such as Xpert MTB/RIF Ultra and Truenat, as the initial diagnostic tool for individuals showing signs and symptoms of TB (Bagcchi, 2023). However, the progress of rapid molecular testing has been sluggish, and its implementation in low- and middle-income countries has been hindered by its prohibitive cost. In 2024, out of 6.4 million diagnosed patients, only 38% were detected using rapid molecular techniques, indicating a need for further advancement and the discovery of additional biomarkers (Carranza et al., 2020; Ghebreyesus, 2024.).

This manuscript provides a comprehensive overview of the strengths, limitations, and clinical applications of several traditional tuberculosis (TB) diagnostic methods. Additionally, it highlights the latest advancements in TB detection technologies, including PET-CT, Xpert MTB/RIF, next-generation sequencing (NGS), and line probe assays, which demonstrate exceptional sensitivity, accuracy, and versatility in various clinical settings. The study also explores the latest TB biomarkers discovered through advanced omics technologies like transcriptomics, metabolomics, and proteomics. These biomarkers are rigorously assessed for their sensitivity, specificity, and practical application, providing key insights into their potential to transform TB diagnosis and management.

## 2 Novel technologies for TB detection

**Figure 2:**
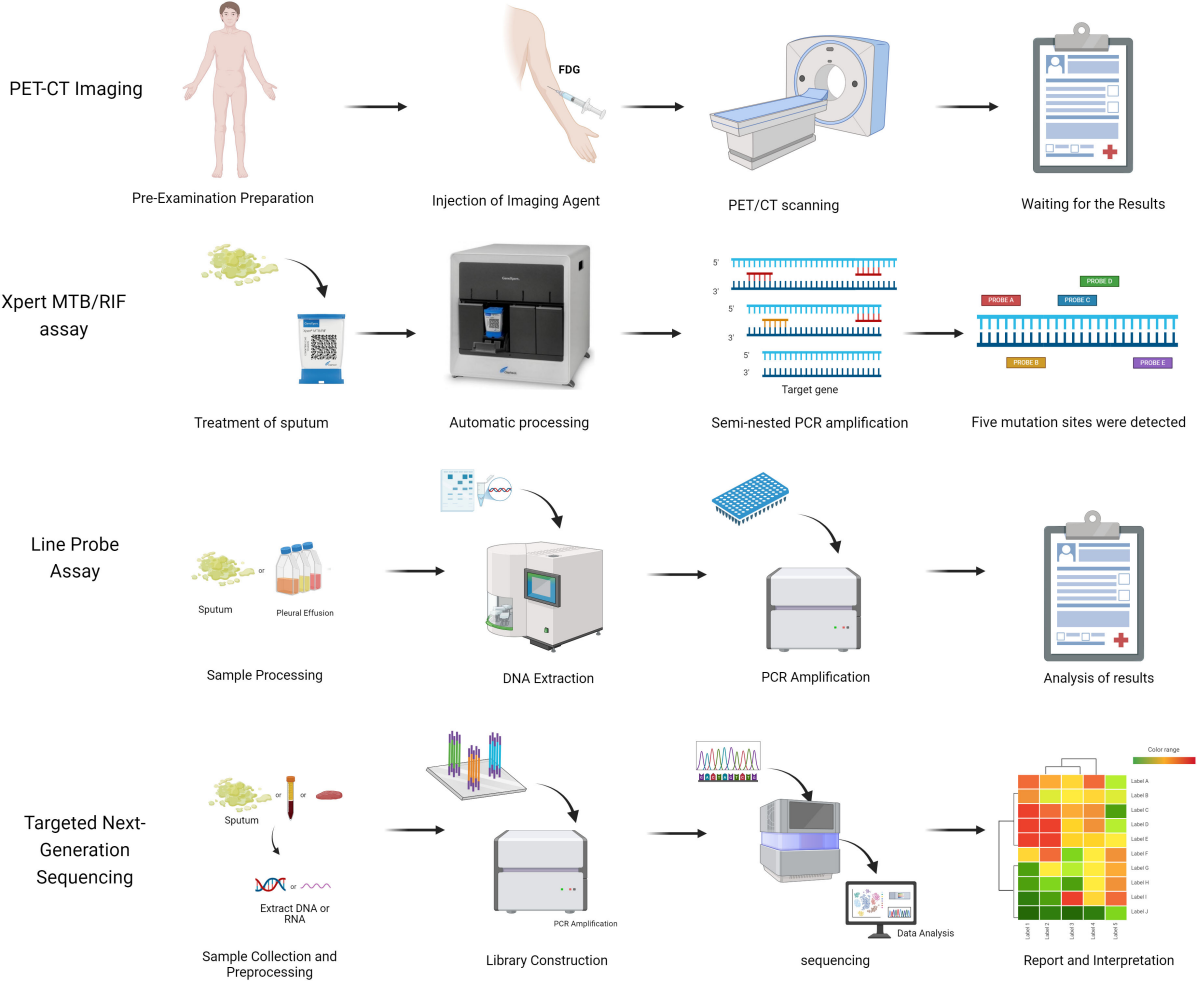
The strategy of novel technologies for TB detection.

In recent years, TB diagnostics have undergone continuous innovation, marked by groundbreaking advancements: The Xpert MTB/RIF nucleic acid amplification technology has notably enhanced diagnostic efficiency through rapid detection and simultaneous analysis of rifampicin resistance genes; AI/ML-based microscopy image analysis systems have achieved automated interpretation of sputum smears, substantially reducing manual errors; PET-CT metabolic-anatomical fusion imaging enables precise localization of active lesions, offering novel pathways for early diagnosis of extrapulmonary TB; and line probe assays (LPAs) facilitate rapid screening of multidrug-resistant tuberculosis (MDR-TB) via targeted analysis of drug resistance gene mutations (**Figure 2**, **Supplementary Table S2**).The synergistic integration of these technologies is propelling TB diagnosis and treatment toward precision medicine and intelligent healthcare paradigms (Khan et al., 2024).

### 2.1 Automated fluorescent microscopy for AI-driven tuberculosis detection

Sputum smear microscopy is the primary diagnostic method for a TB in low-income countries (Hung et al., 2007), with the two most commonly used detection methods being bright-field microscopy and fluorescence microscopy. Manual counting methods require trained inspectors and significant time to search for acid-fast bacilli (AFB) under microscopic vision (Kotei and Thirunavukarasu, 2022). However, implementing an automated system for reading microscopic slides can reduce subjectivity in results and improve the performance of smear microscopy (Mota Carvalho et al., 2023). MetaSystems’ automated fluorescent AFB slide scanner and analyzer represents an innovative commercial solution that integrates computer vision artificial intelligence (AI) with automated digital microscopy (DM) systems (Tomasello et al., 2022). The image acquisition and analysis of AFB slides are performed by the proprietary Metafer software platform, which incorporates a manufacturer-trained deep neural network (DNN) architecture. This advanced AI system, developed through supervised learning methodologies, employs a probabilistic scoring mechanism to accurately identify and classify objects with potential AFB characteristics (Desruisseaux et al., 2024). Among the 496 qualified smears that met the quality control criteria, the MetaSystems platform demonstrated a sensitivity of 97.0% and a specificity of 12.7% when used independently. When positive scans were utilized to assist technologists, the MetaSystems platform achieved a sensitivity of 70.7% and a specificity of 89.0%. Fu et al. further advanced the field by developing an automated microscope system (µ-Scan 1.1) powered by a convolutional neural network (CNN) algorithm, which achieved remarkable performance in detecting acid-fast bacilli (AFB) in tuberculosis sputum smears, with an accuracy of 95.2%, sensitivity of 85.7%, and specificity of 96.9%. In parallel, an innovative cough audio classifier was developed, leveraging AI and ML technologies to analyze cough sounds for tuberculosis detection. When tested in Tanzania, this classifier demonstrated promising results with a sensitivity of 80% and specificity of 90%, underscoring the significant potential of AI in delivering accessible diagnostic solutions, particularly in regions with constrained healthcare infrastructure (Fu et al., 2022).

### 2.2 PET-CT imaging for tuberculosis diagnosis

Chest X-ray, while maintaining its status as the most extensively employed screening modality for suspected pulmonary tuberculosis (PTB) cases, demonstrates significant limitations in detecting early-stage infections and extrapulmonary manifestations owing to its restricted sensitivity and lack of functional imaging capabilities (Feyisa et al., 2023; Sossen et al., 2023). CT and MRI are the preferred imaging techniques for assessing disease presence in specific body sites when sputum is negative or extrapulmonary tuberculosis is suspected (Skoura et al., 2015; Rodriguez-Takeuchi et al., 2019). Positron emission tomography-computed tomography (PET-CT) has emerged as a promising early diagnostic modality for tuberculosis, integrating the functional metabolic imaging capabilities of PET with the high-resolution anatomical details provided by CT, thereby establishing a comprehensive diagnostic framework for tuberculosis detection and evaluation. During the diagnostic procedure, patients undergo intravenous administration of the radioactive tracer 18F-fluorodeoxyglucose (18F-FDG), which serves as a metabolic marker. The PET scanner subsequently detects the positron emissions resulting from the radioactive decay of 18F-FDG, enabling both qualitative visualization and quantitative assessment of metabolic activity in tuberculosis lesions (Lamarca et al., 2019). When combined with CT’s superior anatomical imaging capabilities, this hybrid imaging modality facilitates precise localization and characterization of tuberculosis lesions, significantly enhancing diagnostic accuracy and enabling comprehensive evaluation of disease extent and progression (Bomanji et al., 2020). A comprehensive study conducted by Josef Yayan and colleagues revealed that FDG-PET-CT, while susceptible to interference from other inflammatory diseases, achieved an average diagnostic sensitivity of 82.6% and specificity of 67.3% in tuberculosis detection, demonstrating superior diagnostic performance with significantly higher sensitivity rates compared to conventional methods such as sputum testing (55%), sputum culture (70%), and chest X-ray imaging (72.5%) (Yayan et al., 2024).

### 2.3 Xpert for the detection of tuberculosis

The Xpert MTB/RIF assay (Xpert) and the MTB/RIF Ultra assay (Ultra) are the World Health Organization-recommended rapid molecular detection methods for tuberculosis (Bagcchi, 2023). These assays are utilized for initial diagnostic testing and rifampicin resistance testing in all patients showing signs and symptoms of tuberculosis (Chakravorty et al., 2017). Xpert MTB/RIF is an automated polymerase chain reaction (PCR) test conducted on the GeneXpert platform. Unlike traditional nucleic acid amplification (NAA) tests, Xpert MTB/RIF integrates sample processing, PCR amplification, and detection into a single self-contained test unit (Boehme et al., 2010). All detection steps are self-contained and isolated after sample introduction, with strong *Mtb*-killing ability upon test completion, enabling Xpert to effectively address biosafety concerns during the assay process.

Xpert MTB/RIF Ultra is an enhanced assay that features a newly designed cartridge and can be operated on the same device after a software update (Xie et al., 2024). Compared to Xpert MTB/RIF, Xpert Ultra includes two different multi-copy amplification targets and a larger DNA reaction chamber. The limit of detection (LOD) for TB testing with Ultra is 15.6 colony-forming units (CFUs) per mL in *Mtb*-spiked sputum, which is approximately 8 times higher in sensitivity than Xpert MTB/RIF (Chakravorty et al., 2017). Ultra is expected to improve TB case detection rates, especially in individuals with paucibacillary TB such as those with HIV co-infection, as well as in pediatric patients and those with extrapulmonary TB who typically have lower mycobacterial loads (Wang et al., 2022).

In a comprehensive meta-analysis conducted by Man-Qing Wang and colleagues, systematic evaluation of 187 fourfold tables derived from 72 independent studies demonstrated that Xpert MTB/RIF Ultra achieved an overall pooled sensitivity of 76% and specificity of 95% for pulmonary tuberculosis detection. Furthermore, the assay showed enhanced diagnostic performance in detecting rifampin resistance, with pooled sensitivity reaching 94% and specificity of 97% (Wang et al., 2025).

According to the WHO report, over 80% of rifampicin-resistant TB patients are also resistant to isoniazid, while the majority of rifampicin-resistant patients have MDR-TB (Gandhi et al., 2010). The use of Xpert has greatly enhanced the diagnosis of RIF-R and MDR tuberculosis, leading to a three- to eight-fold rise in global MDR-TB testing between 2010 and 2016 (Penn-Nicholson et al., 2022).Known as the only automated molecular assay of lower complexity, Xpert MTB/XDR is well-suited for a wider range of resistance testing and for use in lower-level laboratory networks (Tabriz et al., 2020; Pillay et al., 2022). This assay is proficient in detecting *M. tuberculosis* complex (MTBC) DNA and mutations linked to resistance against isoniazid, fluoroquinolones (ofloxacin, moxifloxacin, levofloxacin, gatifloxacin), second-line injectable drugs (amikacin, kanamycin, capreomycin), and ethionamide (Su et al., 2017; Pillay et al., 2022).

### 2.4 Line probe assay for the detection of tuberculosis

Line probe assays (LPAs) are a genotyping technique used for detecting drug-resistant *Mtb*, utilizing DNA-DNA hybridization technology and multiple probes to simultaneously identify common resistance mutations (Meaza et al., 2017). Currently endorsed by the WHO for the initial drug resistance screening of sputum smear-positive samples are line probes such as GenoType MTBDRplus, Nipro NTM+MDR-TB, and GenoType MTBDRsl (MacLean et al., 2020; Brankin et al., 2022). GenoType MTBDRplus and Nipro NTM+MDR-TB target mutations in the *rpoB, katG, and inhA* genes, along with the promoter region, to assess resistance to isoniazid and rifampicin (Ferreira Junior et al., 2014). On the other hand, GenoType MTBDRsl examines resistance to ethambutol, quinolones, and second-line injectables by detecting mutations in the *gyrA, rrs, and embB* genes (Brossier et al., 2010; Pinhata et al., 2023).

In a study involving 379 strains and 644 sputum samples (Meaza et al., 2017), the sensitivity and specificity of GenoType MTBDRplus and Nipro NTM+MDR-TB in detecting rifampin resistance ranged from 90.3%-98.2% to 97.8%-98.5% and 92%-96.5% to 97.5%-98.5%, respectively. For isoniazid resistance detection, the sensitivity and specificity were 89.1% and 95.4% to 98.8%-99.4% and 89.6%-94.9% to 97.6%-100%, respectively. Gardee et al. found that compared to phenotypic drug susceptibility testing, GenoType MTBDRsl version 2.0 showed 100% sensitivity for fluoroquinolone resistance and 89.2% sensitivity for second-line injectable drug resistance, with specificities of 98.9% and 98.5%, respectively (Bouzouita et al., 2021). LPAs provide a rapid diagnostic approach for XDR-TB and MDR-TB. However, due to incomplete understanding of the molecular mechanisms of anti-TB drug resistance, LPA targets are limited to common drug resistance mutations (Mindru et al., 2016).

### 2.5 Targeted next-generation sequencing for the diagnosis of tuberculosis

Genotypic drug susceptibility testing methods analyze mutations in the *Mtb* genome associated with drug resistance (Yan et al., 2023). Nucleic acid amplification tests (NAATs) are commonly used for this purpose due to their simplicity and ability to provide results quickly, thereby improving access to drug susceptibility testing (Cantera et al., 2019; Wang et al., 2023). However, NAATs are limited in their scope, targeting only a few known resistance mutations and a select range of drugs.

In contrast, targeted next-generation sequencing (NGS) technology combines gene amplification with high-throughput sequencing to detect resistance to multiple drugs in a single test (Bewicke-Copley et al., 2019; Satam et al., 2023). Targeted NGS can examine entire genes for specific resistance mutations, potentially offering greater accuracy. Furthermore, new targeted NGS assays can identify resistance to novel and repurposed drugs not covered by other molecular tests recommended by the WHO, making them a promising option for comprehensive resistance detection aligned with modern treatment protocols (Schwab et al., 2024).

A recent survey conducted across fifty-three countries, including those on the WHO’s list of high-burden tuberculosis countries, aimed to assess the diagnostic accuracy of targeted NGS for various drugs, including rifampicin, isoniazid, ethambutol, pyrazinamide, streptomycin, injectable drugs (amikacin, capreomycin, and kanamycin), moxifloxacin, and fluoroquinolones like levofloxacin and moxifloxacin (Schwab et al., 2024). The study found that targeted NGS demonstrated an overall sensitivity of 94.1% (95% CrI 90.9 - 96.3) and a specificity of 98.1% (97.0 - 98.9) for drug-resistant tuberculosis testing. In March 2024, the WHO updated its guidelines to include targeted NGS as a recommended tool for rapid diagnostics in tuberculosis detection, highlighting the growing importance and effectiveness of this technology in the fight against drug-resistant TB.

## 3 Potential blood-based biomarkers for tuberculosis diagnosis

Blood-based biomarkers for tuberculosis enable non-invasive, rapid, and highly sensitive diagnosis, with significant clinical potential (Li et al., 2025). Their non-invasive nature simplifies sample collection, avoiding the discomfort of traditional methods like sputum tests or biopsies, especially benefiting children, the elderly, and those unable to provide sputum. Blood-based methods (e.g., ELISA, PCR, mass spectrometry) deliver results in hours, speeding up diagnosis and enabling early treatment. Specific biomarkers (e.g., IP-10, RISK6) are elevated in active tuberculosis, improving sensitivity for low bacterial load cases (Pan et al., 2021). By integrating multiple biomarkers (e.g., cytokines, gene expression, metabolites), the accuracy and specificity of diagnosis can be further enhanced, effectively reducing misdiagnosis and missed diagnoses (**Tables 1**, **2**). Additionally, blood testing technology is well-established, easy to standardize, and suitable for widespread use in resource-limited settings.

**Table 1:** Evaluation of assay performance for Cytokines, Proteins, and Metabolites.

Types	Year	Name	Source	Differentiate	Testing	Validation	Change	Sensitivity	Specificity	AUC	Validation Stage	reference
chemokines	2023	CXCL9	PBMCs	DR-TB vs DS-TB	Luminex Magpix Multiplex Assay system	N/A	UP	N/A	N/A	0.82(p < 0.0001)	Clinical Testing	(Sampath et al., 2023)
chemokines	2023	CXCL10	PBMCs	DR-TB vs DS-TB	Luminex Magpix Multiplex Assay system	N/A	UP	N/A	N/A	0.84(p < 0.0001)	Clinical Testing	(Sampath et al., 2023)
cytokine	2023	IL-27	PBMCs	Early aTB vs HC	mRNA profile	N/A	UP	100%	83%	0.958(p < 0.0010)	Clinical Testing	(Shaukat et al., 2023)
cytokine	2023	IL-24	PBMCs	Early aTB vs HC	mRNA profile	N/A	DOWN	100%	83%	1.00(p < 0.0007)	Clinical Testing	(Shaukat et al., 2023)
cytokine	2021	*Mtb*-specific TNF-α secreting CD38CD27+–CD4 T cells	PBMCs	aTB vs HC	Polychromatic flow cytometry	Flow cytometry	UP	90.16%	96.15%	0.9462(p < 0.0001)	Clinical Testing	(Acharya et al., 2021)
cytokine	2021	IFN-γ and IL - 2	PBMCs	aTB vs HC	ELISA	N/A	UP	87.9%	79.8%	0.859(p < 0.001)	Clinical Testing	(Tan et al., 2021)
cytokine	2019	IP-10	PBMCs	aTB vs HC	ELISA	N/A	UP	74.5%	73%	0.79(p < 0.005)	Preclinical	(Mamishi et al., 2019)
cytokine	2018	GM-CSF	PBMCs	aTB vs LTBI	IGRA	Multiplex immunoassay	DOWN	71%	86%	0.79(p < 0.001)	Preclinical	(Balcells et al., 2018)
cytokine	2018	IL-2	PBMCs	aTB vs HC	IGRA	Multiplex immunoassay	UP	73.5%	85%	0.79(p < 0.0001)	Preclinical	(Balcells et al., 2018)
Protein	2024	FCGR3B, FETUB, LRG1, ADA2, CD14 and SELL	Plasma	aTB vs HC	tandem mass spectrometry	proximity extension assays	UP	90.6%	90.0%	0.972(p < 0.0001)	Clinical Testing	(Schiff et al., 2024)
Protein	2022	ALB, HP, OAF and RBP4	Plasma	untreated TB vs cured TB	Data-independent acquisition	N/A	UP/DOWN/DOWN/UP	91.4%	94.3%	0.963(p < 0.001)	Clinical Testing	(Lu et al., 2022)
Protein	2021	CXCL1	PBMCs	aTB vs HC	Microarray gene expression	ELISA	UP	100.0%	98.4%	0.999(p < 0.0001)	Clinical Testing	(Koyuncu et al., 2021)
Protein	2021	CXCL1	PBMCs	aTB vs LTBI	Microarray gene expression	ELISA	UP	94.5%	88.8%	0.972(p < 0.0001)	Clinical Testing	(Koyuncu et al., 2021)
Protein	2021	CXCL1	PBMCs	aTB vs non-TB	Microarray gene expression	ELISA	UP	90.9%	71.4%	0.921(p < 0.0001)	Clinical Testing	(Koyuncu et al., 2021)
Protein	2020	sCD14, PGLYRP2 and FGA	Serum	MDR-TB vs DS-TB	DIA combined with PRM	N/A	DOWN/UP/UP	81.2%	90%	0.934(p < 0.001)	Clinical Testing	(Chen et al., 2020)
Protein	2020	CFHR5, LRG1, LBP, SAA1, and CRP	Plasma	aTB vs HC	high-resolution mass spectrometry	ELISA or Luminex array	UP	N/A	N/A	0.93(p < 0.001)	Preclinical	(Garay-Baquero et al., 2020)
Protein	2020	G-CSF, C3b/iC3b, procalcitonin, IP-10, PDGF-BB	Plasma	aTB vs HC	Luminex multiplex platform	customized, focused panel array	UP	72.7%	90.5%	0.93(p < 0.001)	Preclinical	(Garay-Baquero et al., 2020)
Metabolites	2023	Meso-hydroxyheme and itaconic anhydride	Plasma	pre-XDR and XDR-TB vs pan-susceptible group	UHPLC-ESI-QTOF-MS/MS	N/A	UP	100%	100%	1.00(p < 0.001)	Clinical Testing	(Chaiyachat et al., 2023)
Metabolites	2021	Cer (d18:1/24:0), CerP (d18:1/20:3), LPE (0:0/22:0), LPA (0:0/16:0), and LPA (0:0/18:0)	Plasma	cured TB vs untreated TB	UPLC-MS/MS	N/A	UP	N/A	N/A	1.00(p < 0.0001)	Preclinical	(Chen et al., 2021)
Metabolites	2021	5-hydroxyindoleacetic acid, isoleucyl-isoleucine, heptadecanoic acid, indole acetaldehyde, 5-ethyl-2,4-dimethyloxazole, and 2-hydroxycaproic acid, unknown 71	Plasma	aTB vs HC	GC-TOF MS and UHPLC-QE-MS	N/A	UP/UP/DOWN/DOWN/DOWN/DOWN/DOWN	N/A	N/A	0.97 (p < 0.001)	Preclinical	(Jiang et al., 2021)
Metabolites	2021	PC (12:0/22:2), PC (16:0/18:2), cholesteryl ester (20:3), and sphingomyelin (d18:0/18:1)	Plasma	aTB vs HC	UPLC-MS/MS	10-fold cross-validation	DOWN/DOWN/UP/DOWN	92.9%	82.4%	0.934(p < 0.001)	Preclinical	(Han et al., 2021)
Metabolites	2019	Choline, glycine, serine, threonine and homoserine	Plasma	aTB vs TB-DM	LC-MS/MS	N/A	DOWN	N/A	N/A	0.967(p < 0.001)	Preclinical	(Vrieling et al., 2019)
Metabolites	2018	PG (16:0_18:1), Lyso-PI (18:0) and Ac1PIM1 (56:1)	Plasma	aTB vs HC	High-resolution metabolomics	N/A	UP	N/A	N/A	0.97(p < 0.001)	Preclinical	(Collins et al., 2018)

**Table 2:** Evaluation of assay performance for LncRNAs, MicRNAs and CirRNAs.

Types	Year	Name	Source	Differentiate	Testing	Validation	Change	Sensitivity	Specificity	AUC	Validation Stage	reference
LncRNA	2022	TCONS_00001838 and n406498 + EHR	PBMCs	aTB vs HC	Affymetrix HTA2.0 array	qRT-PCR	DOWN	81%	73%	0.86(p < 0.001)	Preclinical	(Chen et al., 2022)
LncRNA	2022	NORAD	Plasma	aTB vs HC	qRT-PCR	N/A	UP	80.0%	89.4%	0.918(p < 0.0001)	Preclinical	(Sun et al., 2022)
LncRNA	2021	NONHSAT078957.2, NONHSAT067134.2, NONHSAT101518.2, NONHSAT148822.1	Plasma	aTB vs HC	Data analysis	qRT-PCR	DOWN	N/A	N/A	0.8994, 0.8725, 0.9502, 0.7080(p < 0.001)	Preclinical	(Fang et al., 2021)
LncRNA	2021	n344917 + EHR	Plasma	aTB vs HC	qRT-PCR	Prediction model	DOWN	88.98%	86.43%	0.88(p < 0.0001)	Preclinical	(Meng et al., 2021)
LncRNA	2020	uc.48+ and NR_105053	Plasma	untreated TB vs cured TB	lncRNA microarray	qRT-PCR	DOWN	90.00%	86.36%	0.945(p < 0.001)	Preclinical	(Li et al., 2020)
LncRNA	2020	ENST00000497872, n333737, and n335265	PBMCs	aTB vs HC	lncRNA microarray and qRT-PCR	Prediction model	DOWN/DOWN /UP	86%	82%	0.89(p < 0.001)	Preclinical	(Hu et al., 2020)
LncRNA	2019	LOC152742	Sputum	aTB vs HC	qRT-PCR	qRT-PCR	UP	91.02%	88.62%	0.914(p < 0.0001)	Preclinical	(Wang et al., 2019)
LncRNA	2019	LOC152742	Plasma	aTB vs HC	qRT-PCR	qRT-PCR	UP	93.61%	86.21%	0.92 (p < 0.0001)	Preclinical	(Wang et al., 2019)
LncRNA	2017	NR 038221, NR003142, ENST00000570366, and ENST00000422183	Plasma	aTB vs HC	lncRNA microarray	qRT-PCR	UP/UP/UP/DOWN	79.2%	75%	0.845(p < 0.001)	Preclinical	(Chen et al., 2017)
MicRNA	2024	miR-29a	Sputum, PBMCs, cerebrospinal fluid and plasma	aTB vs HC	Systematic review	N/A	UP	82%	82%	0.89(p < 0.001)	Preclinical	(He et al., 2024)
MicRNA	2023	hsa-miR-425-5p, hsa-miR-4523	Plasma	LNTB vs LTBI	qRT-PCR	N/A	UP\DOWN	90.5%	81%	0.907(p < 0.001)	Preclinical	(Massi et al., 2023)
MicRNA	2021	miR-185-5p	exosomes	aTB vs HC	whole transcriptome sequencing	qRT-PCR	UP	50%	0.9375%	0.750 (p < 0.01)	Preclinical	(Kaushik et al., 2021)
MicRNA	2019	miR-20a, miR-20b, miR-26a, miR-106a, miR-191, miR-486	Plasma	PTB vs HC	Affymetrix Genechip miRNA 4.0 Array	qRT-PCR	UP	89%	89%	0.97(p < 0.001)	Preclinical	(Hu et al., 2019)
MicRNA	2019	miR-146a, miR-125b-5p and MTBmiR-5	Plasma	PTB vs HC	Ion Torrent PGM platform	qRT-PCR	UP	100%	56.67%	0.799(p < 0.001)	Preclinical	(Chakrabarty et al., 2019)
MicRNA	2019	miR-146a, miR-125b-5p and MTBmiR-5	Plasma	EPTB vs HC	Ion Torrent PGM platform	qRT-PCR	UP	85.29%	56.67%	0.688(p = 0.007)	Preclinical	(Chakrabarty et al., 2019)
MicRNA	2019	miR-892b	PBMCs	aTB vs HC	Integrated bioinformatics	qRT-PCR	DOWN	55%	90%	0.77(p < 0.05)	Preclinical	(Zhang et al., 2019)
MicRNA	2019	miR-582-5p	PBMCs	aTB vs HC	Integrated bioinformatics	qRT-PCR	UP	40%	95%	0.70(p < 0.01)	Preclinical	(Zhang et al., 2019)
MicRNA	2017	miR-199b-5p	PBMCs	aTB vs HC	Integrated bioinformatics	qRT-PCR	UP	50%	80%	0.71(p < 0.001)	Preclinical	(Zhang et al., 2019)
circRNA	2020	hsa_circ_0001380	Plasma	aTB vs HC	circRNA−sequencing (seq)	qRT-PCR	DOWN	93.75%	87.50%	0.9502(p < 0.001)	Preclinical	(Luo et al., 2020)
circRNA	2020	hsa_circ_0028883	Plasma	aTB vs HC	Data retrieval and discrepancy analysis	qRT-PCR	UP	N/A	N/A	0.773(p < 0.01)	Preclinical	(Zhang et al., 2020)
circRNA	2019	hsa_circRNA_101128	PBMCs	aTB vs HC	Arraystar Microarray	qRT-PCR	UP	N/A	N/A	0.817(p < 0.001)	Preclinical	(Fu et al., 2019)
circRNA	2019	hsa_circRNA_103017	Plasma	aTB vs HC	Arraystar Microarray	qRT-PCR	UP	N/A	N/A	0.870(p < 0.001)	Preclinical	(Fu et al., 2019)
circRNA	2018	hsa_circRNA_059914	PBMCs	aTB vs HC	Arraystar Microarray	qRT-PCR	UP	N/A	N/A	0.821(p < 0.001)	Preclinical	(Fu et al., 2019)
circRNA	2018	hsa_circRNA_001937	PBMCs	aTB vs HC	Human CircRNA Array v1.0	qRT-PCR	UP	85%	77.5%	0.873(p < 0.0001)	Preclinical	(Huang et al., 2018a)
circRNA	2018	hsa_circRNA_103571	Plasma	aTB vs HC	Arraystar Human Circular RNA Microarray V2	qRT-PCR	DOWN	N/A	N/A	0.838	Preclinical	(Yi et al., 2018)
circRNA	2018	hsa_circ_0001204, hsa_circ_0001747	Plasma	aTB vs HC	Human CircRNA Array V2.0	qRT-PCR	UP	86.21%	89.17%	0.928(p < 0.001)	Preclinical	(Huang et al., 2018c)
circRNA	2018	hsa_circ_0001953; hsa_circ_0009024	Plasma	aTB vs HC	Arraystar circRNA Microarray	qRT-PCR	UP	72.50%	96.00%	0.915(p < 0.001)	Preclinical	(Huang et al., 2018b)

### 3.1 Cytokine-based biomarkers for tuberculosis diagnosis

Adaptive immunity plays a critical role in the progression of tuberculosis, a fact well-established and extensively documented (Jasenosky et al., 2015; de Martino et al., 2019). In the early stages of *Mtb* infection, cell-mediated immune responses are vital for containing the pathogen within a localized lung area (Li et al., 2019; Shaukat et al., 2023). In a study conducted by Muthya Pragun Acharya et al., 245 individuals were recruited and categorized into groups including PTB, EPTB, LTBI, healthy controls (HCs), cured tuberculosis (CTB), and sick controls (SCs). Host immune biomarkers in peripheral blood mononuclear cells were identified using multicolor flow cytometry in a cohort of 56 subjects. The clinical performance of these biomarkers was subsequently evaluated in a blind validation cohort of 165 subjects using whole blood (Acharya et al., 2021). The results indicated that the frequencies of cytokine-secreting *Mtb*-specific CD4 T cells with the CD38+CD27– phenotype clearly distinguished individuals with active tuberculosis from those without the disease. Among the cytokines tested, tumor necrosis factor-α (TNF-α) secretion from CD38+CD27-CD4+ T cells upon stimulation with ESAT6/CFP10 peptides demonstrated the best diagnostic accuracy, with a cutoff of 9.91% (exploratory results: 96.67% specificity, 88.46% sensitivity; validation results: 96.15% specificity, 90.16% sensitivity). Furthermore, this T cell subset could differentiate between treatment-naive TB patients and individuals who had been successfully treated for TB post-anti-TB therapy.

The persistent spread of drug-resistant TB remains one of the most urgent and formidable challenges confronting the global efforts to control TB. In 2023, Pavithra Sampath and colleagues identified two potential biomarkers capable of distinguishing between drug-sensitive and drug-resistant tuberculosis (Sampath et al., 2023). The study population included groups of HCs, individuals with LTBI, drug-sensitive tuberculosis (DS-TB), and drug-resistant tuberculosis (DR-TB), with 40 participants in each group, while those with other infections and comorbidities such as diabetes, HIV, HCV, and HBV were excluded from the study. The experiment initially confirmed that drug-resistant tuberculosis was linked to increased levels of chemokines in plasma using the Luminex Magpix multiplex detection system (Bio-Rad, Hercules, CA). Following this, CXCL10 and CXCL9 exhibited statistically significant differences across all four groups according to ROC analysis of individual variables. The findings were further validated through random forest (RF) analysis (**Table 1**).

### 3.2 Proteomics-based screening for diagnostic biomarkers of tuberculosis

Proteomics has emerged as a crucial tool for comprehensively analyzing cellular and organismal processes related to disease and its progression at the protein level (Kavallaris and Marshall, 2005; Al-Amrani et al., 2021). By profiling proteins, proteomics helps uncover the intricate connections among different cellular pathways, complementing both genomic studies and traditional biochemical methodologies (Pandey and Ghosh, 2024; Schiff et al., 2024).

Up to now, the majority of mass spectrometry-based (MS-based) proteomics studies have removed numerous enriched protein components from plasma, resulting in the loss of biologically significant proteins. Consequently, candidate host proteins serving as TB biomarkers commonly exhibit high sensitivity but inadequate specificity. In a study led by Hannah F. Schiff et al., an optimized non-depletion untargeted proteomics method was employed to enhance the coverage of numerous enriched proteins. This approach enables the identification of new markers with both high sensitivity and specificity for tuberculosis (Schiff et al., 2024). Plasma samples from 11 aTB patients and 10 HCs in South Africa and Peru were initially subjected to proteomics analysis. Subsequently, through bioinformatics analysis that employed linear modeling and whole-gene correlation network analysis (WGCNA), a total of 118 differentially expressed proteins were identified. An independent patient cohort from the United Kingdom was later employed to validate the diagnostic potential of MS-identified plasma biomarkers, which included 32 patients with active TB and 30 individuals in a healthy control group. The final 6-protein marker combination, comprising FCGR3B, FETUB, LRG1, ADA2, CD14, and SELL, effectively differentiated patients with aTB from HCs and other infections (ORI) with high sensitivity and specificity. The AUC for TB and HCs was 0.972, with a sensitivity of 90.6% and a specificity of 90.0%, while the AUC for TB and ORI was 0.930, with a sensitivity of 90.6% and a specificity of 80.8% (**Table 1**).

Treatment for newly diagnosed TB consists of an intensive period of two months followed by a continuation period of four months. However, the absence of established criteria and biomarkers remains a challenge in effectively diagnosing cured TB. Qiqi Lu et al. utilized data-independent acquisition (DIA) to analyze the plasma protein expression profiles of TB patients at different treatment stages, which encompassed 35 newly diagnosed TB patients without treatment (group TB0), 35 TB patients after 2-month intensive-phase treatment (group TB2), 35 cured TB patients after 6-month intensive plus continuation phase treatment (group TB6), and 35 healthy controls (group HC) for comparison (Lu et al., 2022). Subsequent analysis of gene ontology (GO) function and Kyoto Encyclopedia of Genes and Genomes (KEGG) pathways indicates possible coagulation dysfunction, along with disruptions in vitamin and lipid metabolism, during tuberculosis treatment. By leveraging Machine Learning and Support Vector Machine (SVM) method, we ultimately identified four intersecting proteins (albumin [ALB, Swissprot: A0A0C4DGB6], haptoglobin [HP, Swissprot: P00738], Out at first protein homolog [OAF, Swissprot: E9PJ29], and retinol-binding protein 4 [RBP4, Swissprot: P02753]) as promising biomarkers for assessing the efficacy of pulmonary tuberculosis treatment. The efficacy assessment model, utilizing the four proteins, achieved an AUC of 0.963 with a sensitivity of 91.4% and specificity of 94.3% in distinguishing between TB0 and TB6 groups, while also demonstrating an AUC of 0.971, sensitivity of 88.6%, and specificity of 94.3% in distinguishing TB0 and HC groups.

### 3.3 Metabolomics–based screening for diagnostic biomarkers of tuberculosis

Metabolomics enables the quantitative profiling of high-throughput metabolite molecules (Cao et al., 2020). Through the analysis of metabolite changes, metabolomics can pinpoint specific differentially expressed metabolites that act as biomarkers for diagnostics, disease differentiation, and monitoring the effectiveness of treatment (Vinayavekhin et al., 2010).

The host plasma is abundant in lipids, which constitutes the primary source of nutrition for the growth and reproduction of *Mtb*. The high-throughput detection of alterations in the entire lipid metabolome of the host caused by *Mtb* infection was conducted by Jia-Xi Chen et al. using ultra-performance liquid chromatography-tandem mass spectrometry (UPLC-MS/MS) technology (Chen et al., 2021). The Orthogonal Partial Least Squares-Discriminant Analysis (OPLS-DA) model revealed that lipid metabolites between the TB0 group and the HC group were distinctly distinguishable, with 163 differential lipids identified. Additionally, comparisons among the TB0, TB2, and TB6 groups highlighted 25 lipid metabolites as differential. KEGG pathway analysis showed that the plasma from the TB0 and HC groups displayed differences in metabolic pathways linked to glycerophospholipid and sphingolipid metabolism and autophagy, with a noticeable enrichment in linolenic acid and arachidonic acid metabolic pathways as treatment progressed. Together, these metabolites constituted an efficacy evaluation model that accurately distinguished patients in the TB6 group from those in the TB0 group, achieving a perfect area under the curve (AUC) of 1.000. Specifically, the two lysophosphatidic acids, LPA (0:0/16:0) and LPA (0:0/18:0), were instrumental in differentiating cured and active TB patients, exhibiting an AUC of 1, with both sensitivity and specificity reaching 100%.

Due to the overlapping thresholds in drug susceptibility testing for anti-TB drugs, identifying pre-extensively (pre-XDR) and extensively drug-resistant tuberculosis (XDR-TB) poses a significant challenge. Pratchakan Chaiyachat and colleagues conducted a metabolomic analysis of one hundred and fifty *Mtb* isolates using ultra-high performance liquid chromatography coupled with electrospray ionization-quadrupole-time of flight-mass spectrometry (UHPLC-ESI-QTOF-MS/MS) (Chaiyachat et al., 2023). This analysis included fifty-four pre-XDR, sixty-three XDR-TB, and thirty-three pan-susceptible (pan-S) isolates. Twelve metabolic markers exhibiting the most significant differences between groups were identified. Notably, meso-hydroxyheme and itaconic anhydride demonstrated the ability to accurately classify the resistance status of a sample with 100% sensitivity and specificity. In addition, specific metabolites were identified in *Mtb* isolates resistant to ethionamide (ETO) and ethambutol (ETH).

### 3.4 LncRNAs-based biomarkers for tuberculosis diagnosis

**Figure 3:**
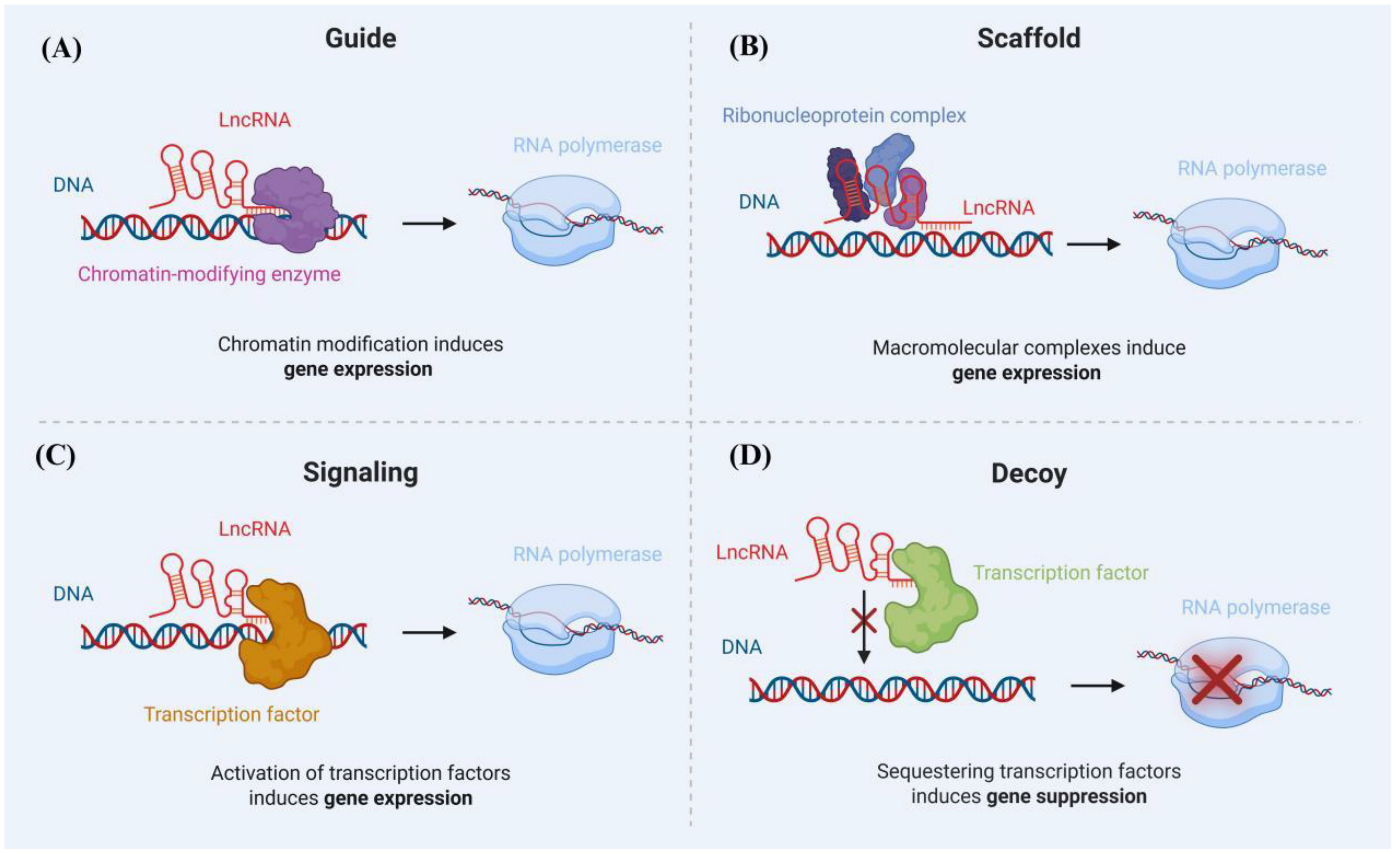
Mechanisms of lncRNA Action: **(A)** LncRNAs recruit chromatin-modifying enzymes to specific gene loci, whereby the modulation of chromatin status results in the activation or suppression of nearby genes. **(B)** LncRNAs engage in the formation of RNA-protein complexes (RNPs), facilitating either the promotion or repression of transcription. **(C)** LncRNAs recruit transcriptional machinery proteins to nearby target gene sites in order to enhance their transcriptional activity. **(D)** LncRNAs act as a decoy for the transcription factor, contributing to the repression of certain pro-apoptotic genes.

Long non-coding RNAs (lncRNAs) are single noncoding RNA transcripts longer than 200 nucleotides, which are crucial elements in regulating gene expression (**Figure 3**). There is a growing body of evidence indicating that blood lncRNA expression profiles are closely linked to tuberculosis (TB), suggesting their potential as noninvasive biomarkers for TB detection (Wei et al., 2017; Mattick et al., 2023).

In research carried out by Zhong-liang Chen et al., plasma lncRNAs were scrutinized through microarray analysis, with a special focus on investigating the potential diagnostic significance of lncRNAs in TB (Chen et al., 2017). Through a thorough analysis, it was revealed that there were 511 differentially expressed lncRNAs (163 up-regulated and 348 down-regulated) as well as 411 differentially expressed mRNAs (127 up-regulated and 284 down-regulated) when comparing individuals with TB to healthy controls. GO, KEGG, and coding-noncoding co-expression (CNC) analyses revealed that the differentially expressed lncRNAs predominantly played a role in regulating alpha-beta T cell activation and the T cell receptor signaling pathway. Six lncRNAs (NR_038221, NR_003142, ENST00000568177, ENST00000570366, ENST00000422183, and ENST00000449589) were chosen for qPCR validation in 52 TB patients and healthy controls. However, there were no significant differences in the expression levels of ENST00000568177 and ENST00000449589 between TB patients and healthy control subjects. The final diagnostic model consisted of four distinct lncRNAs: NR_038221, NR_003142, ENST00000570366, and ENST00000422183, achieving an AUC of 0.845 with a sensitivity of 79.2% and a specificity of 75%. Furthermore, the lncRNA-mRNA-miRNA ceRNA network was developed to predict potential interactions between 85 mRNAs and 404 miRNAs with the identified lncRNAs (**Table 2**).

Plasma lncRNA could potentially serve as a biomarker for accurately evaluating the recovery status of tuberculosis. Zhi-Bin Li et al. utilized lncRNA microarray analysis to identify differentially expressed plasma lncRNAs in untreated and cured TB individuals. They confirmed the expression levels of these lncRNAs using qPCR (Li et al., 2020). The findings revealed significant differences in the expression of lncRNAs uc.48+ and NR_105053 between untreated and cured tuberculosis groups. These lncRNAs were used to establish a predictive model for tuberculosis recovery. The model demonstrated a sensitivity of 90.00%, specificity of 86.36%, and an AUC value of 0.945. These lncRNAs could serve as biomarkers to differentiate between untreated TB patients and those who have been cured. Furthermore, the study predicted target genes of uc.48+ and NR_105053 by constructing co-expression networks between coding and non-coding genes, as well as an mRNA-lncRNA-miRNA interaction network.

### 3.5 MicRNAs–based biomarkers for tuberculosis diagnosis

MicroRNAs (miRNAs) are small non-coding RNAs, typically 18 to 24 nucleotides in length, They regulate gene expression at the post-transcriptional level and play a crucial role in various biological processes, including immune responses (Ying et al., 2008). Exosomal miRNAs have emerged as promising biomarkers, and in a study by Xuejiao Hu et al., they were combined with electronic health records (EHRs) for tuberculosis diagnosis (Gao et al., 2021). In the initial phase of the study, microarrays were utilized to analyze an exploratory cohort consisting of 11 active TB patients (7 PTB and 4 tuberculosis meningitis) and 8 HCs, aiming to identify differentially expressed exosomal miRNAs. Eleven candidate miRNAs, with miR-486 among them, were chosen from a pool of 102 differentially expressed exosomal miRNAs. Finally, six exosomal miRNAs (miR-20a, miR-20b, miR-26a, miR-106a, miR-191, and miR-486) were found to be differentially expressed in TB patients through qRT-PCR analysis. Subsequently, miRNAs and EHRs were employed to construct diagnostic models for PTB and Tuberculous Meningitis (TBM) in the selection cohort utilizing the Support Vector Machine (SVM) algorithm. The integrated “EHR+miRNA” model exhibited superior performance compared to using EHR data or miRNA data independently, achieving a diagnostic sensitivity of 0.94 and a specificity of 0.95 for TBM. For PTB, the sensitivity was 0.89, with a corresponding specificity (**Table 2**).

In a separate study, Muhammad Nasrum Massi and colleagues identified distinct expression patterns of miR-425-5p and miR-4523 in patients with active PTB, LTBI, and lymph node tuberculosis (LNTB) (Massi et al., 2023). The total study sample consisted of 23 patients with active PTB, 21 patients with LTBI, 21 patients with EPTB, and 25 HCs. The levels of hsa-miR-425-5p and hsa-miR-4523 in blood samples from various populations were quantified using RT-qPCR. The level of hsa-miR-425-5p miRNA expression in LNTB was found to be higher than that observed in LTBI. Additionally, the expression of hsa-miR-4523 miRNA was notably lower in PTB and LNTB compared to LTBI. ROC analysis of a single sample revealed that only mir-4523 had the capability to distinguish between LTBI and HCs, showcasing an AUC of 0.829 (**Table 2**).

### 3.6 CirRNAs–based biomarkers for tuberculosis diagnosis

**Figure 4:**
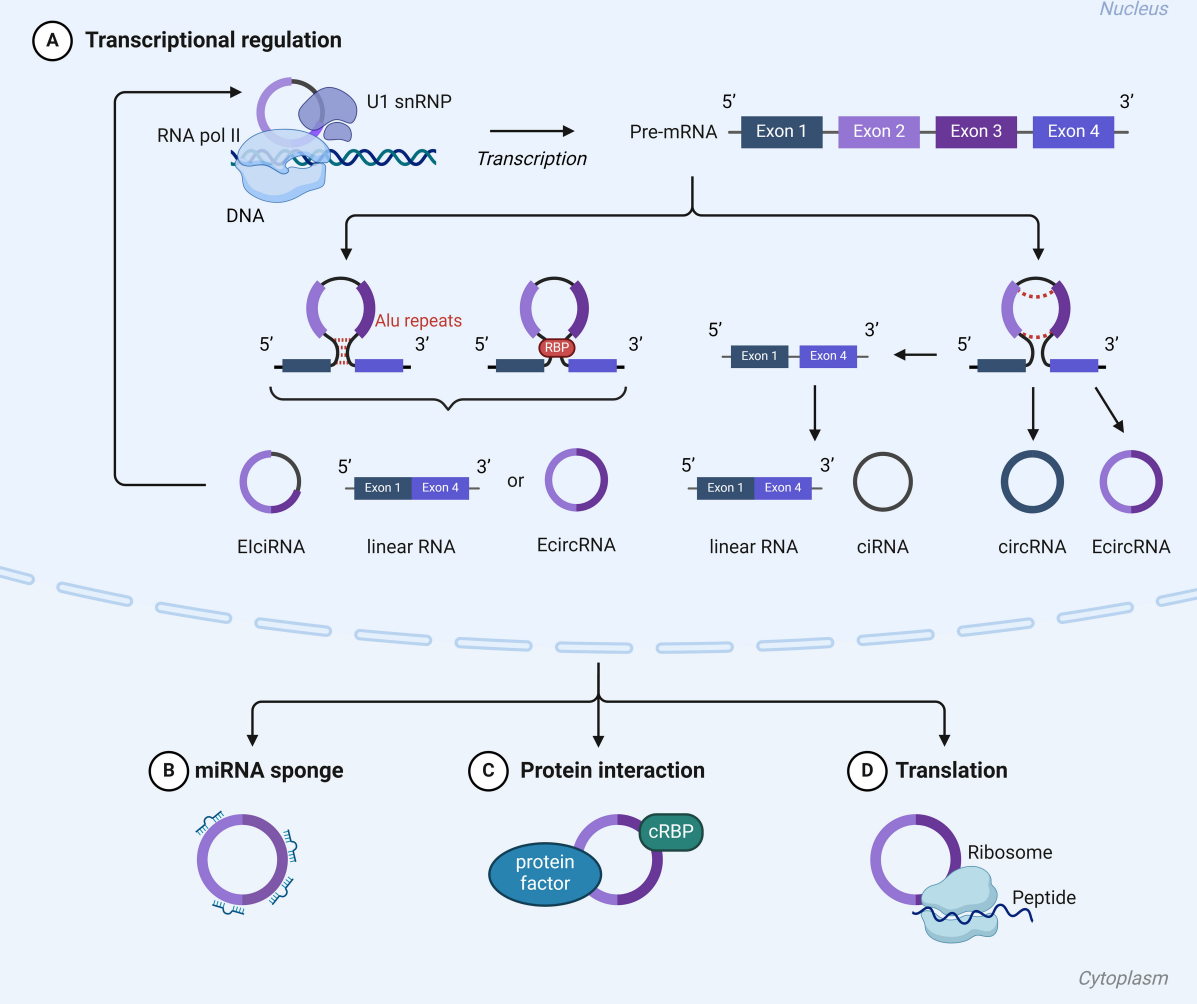
Mechanisms of action of cirRNA: **(A)** CircRNAs have the capability to directly interact with transcription complexes, thereby influencing the expression of parental genes. **(B)** CircRNAs have the ability to act as miRNA sponges. **(C)** circRNAs can interact with circRNA binding proteins (cRBPs) modulate their functions. **(D)** CircRNAs have the ability to encode peptides and proteins.

Circular RNAs (circRNAs) represent a unique class of RNA characterized by the covalent linkage of their 3’ and 5’ ends, forming a closed-loop structure (Ruiz Esparza Garrido and Velázquez Flores, 2023) (**Figure 4**). Unlike linear RNAs, circRNAs, with their covalently closed loops, exhibit heightened resistance to RNase degradation, making them preferentially enriched during sample processing and superior candidates for molecular diagnostic biomarkers compared to other RNA types (Qu et al., 2015).

In a study by Zi-Kun Huang et al., differential circRNA expression was analyzed using microarray assay in three patients diagnosed with aTB and three HCs matched in terms of age and gender. A total of 37 circRNAs were identified as being differentially expressed between the two groups, comprising 13 up-regulated circRNAs and 24 down-regulated circRNAs. The three most significantly up-regulated and down-regulated circRNAs were chosen for validation using qRT-PCR in an independent cohort comprising 40 TB patients and 40 healthy controls. The findings suggested that hsa_circRNA_001937, hsa_circRNA_009024, and hsa_circRNA_005086 were elevated, while hsa_circRNA_102101, hsa_circRNA_104964, and hsa_circRNA_104296 were decreased in TB patients compared to the normal control group. The AUCs of the six candidate circRNAs were all above 0.750, with hsa_circRNA_001937 attaining the highest AUC of 0.873, outperforming the other five circRNAs in the study. hsa_circRNA_001937 was subsequently examined in a separate cohort comprising 115 TB patients, 40 pneumonia patients, 40 chronic obstructive pulmonary disease (COPD) patients, 40 lung cancer patients, and 90 control subjects. In the new cohort, the AUC of hsa_circRNA_001937 was 0.850, with sensitivity and specificity rates of 72.2% each; the expression level of hsa_circRNA_001937 in this cohort was significantly higher than that in patients with pneumonia and lung cancer (**Table 2**).

A thorough analysis integrating bioinformatics and molecular biology revealed that hsa_circ_0028883 holds promise as a potential biomarker for the diagnosis of active tuberculosis (Zhang et al., 2020). The study collected gene expression datasets for circRNA (GSE117563 and GSE106953), microRNA (miRNA, dataset GSE29190), and mRNA (GSE54992) from the Gene Expression Omnibus (GEO) database. A competing endogenous RNA (ceRNA) network was constructed based on potential interactions between circRNA, miRNA, and mRNA (Xie et al., 2018). GO and KEGG pathway analyses were used to predict their biological functions. To validate the results, qRT-PCR was performed to measure hsa_circ_0028883 and hsa-miR-409-5p levels in 20 active TB patients and 20 healthy donors. An ROC curve was then generated to evaluate hsa_circ_0028883’s diagnostic value. We discovered that hsa_circ_0028883 exhibited an impressive AUC value of 0.773 (**Table 2**).

## 4 Discussion

Significant progress has been made in tuberculosis diagnosis over the past few decades, transitioning from culture-based methods to faster, more accurate tests that are less labor-intensive and time-consuming, without the need for sophisticated biosafety labs (Walzl et al., 2018; Xie et al., 2018). Nonetheless, achieving the 2030 targets outlined in the World Health Organization’s End TB Strategy will necessitate ongoing technological advancements (Gupta-Wright and Denkinger, 2024).

Beyond improving diagnostic methods, it is crucial to collect and analyze biologic treatment data for TB patients during their treatment (Ahuja and Zaheer, 2025) (**Supplementary Table S3**). This involves documenting TB exposure history, symptom checks, and comorbidities. Key diagnostic tools like skin testing/IGRA, Xpert MTB/RIF, sputum culture, and chest X-rays should be carefully recorded. Additionally, the use of biologic agents such as TNF-α inhibitors and chemopreventive therapy, along with standard treatment protocols and immunosuppression data, must be thoroughly tracked (Naidu et al., 2023). TNF-α is a key biological agent for treating autoimmune diseases and a critical factor in maintaining the structure and function of tuberculous granulomas. Its inhibition can destabilize granulomas, leading to the reactivation of LTBI or the rapid progression of new *Mtb* infections to active tuberculosis. In terms of diagnosis, TNF-α inhibitors can suppress delayed-type hypersensitivity (DTH), resulting in a false-negative rate of up to 50-70% in the TST (de Oliveira Magalhães et al., 2024). Although the IGRA is not affected by DTH, long-term TNF-α inhibition may reduce IFN-γ release levels, increasing the risk of false negatives by 20-30%. Therefore, when using these drugs, it is essential to combine molecular testing (e.g., Xpert MTB/RIF) and imaging assessments to mitigate the interference of immune suppression on traditional diagnostic methods. The impact of chemopreventive therapy on tuberculosis diagnosis is equally significant. It may reduce the activity or quantity of *Mtb*, affecting the sensitivity of bacterial culture or molecular testing and leading to false-negative results. Additionally, chemopreventive therapy may modulate immune responses, compromising the accuracy of TST or IGRA and resulting in false negatives. When evaluating treatment efficacy or monitoring for relapse, chemopreventive therapy may also interfere with LTBI diagnosis. Therefore, interpreting diagnostic results requires integrating clinical context with other testing methods, taking into account the influence of chemopreventive therapy (Prakash Babu et al., 2023).

Furthermore, future research efforts should prioritize the development and application of cutting-edge technologies with the potential to transform TB diagnosis, such as Spatial CITE-seq for spatially resolved single-cell analysis, multimodal tri-omics for comprehensive molecular profiling, and perturb-DBiT for high-throughput functional genomics (Zhang et al., 2024). These innovative approaches hold promise for unraveling the complex mechanisms of TB pathogenesis and enabling more precise, early-stage detection. Despite the remarkable strides in tuberculosis (TB) diagnosis, several challenges persist. The prohibitive costs associated with many cutting-edge diagnostic technologies act as a significant barrier to their implementation in low-resource settings. Additionally, the detection of certain biomarkers necessitates sophisticated laboratory infrastructure and technical expertise, which are often unavailable in primary healthcare facilities. Future research endeavors should focus on elucidating the clinical utility of biomarkers, refining detection methodologies, and reducing costs to enhance accessibility (Baysoy et al., 2024).
