# Supplementary material for: Recent progress in tuberculosis diagnosis: insights into blood-based biomarkers and emerging technologies
Source: Front Cell Infect Microbiol. 2025 May 8;15:1567592. doi: 10.3389/fcimb.2025.1567592 (PMC12094917; doi:10.3389/fcimb.2025.1567592)
Supplement: Supplementary file 1 [file Table1.docx]

### Table S1. Analysis of the Characteristics of Existing Traditional Technologies

| Diagnostic Techniques | Advantages | Limitations | Characteristics | Applicability |
| --- | --- | --- | --- | --- |
| Sputum smear microscopy | Cost-effectiveness, Rapid results, High specificity | Low sensitivity,  Operator dependency, Inability to detect drug resistance | Direct pathogen observation, Applicability for large-scale screening | Active pulmonary tuberculosis |
| Sputum culture | Low cost, relatively simple operation, Accurate pathogen identification | Long result turnaround time, susceptible to various factors | Isolation of viable bacteria | Active pulmonary tuberculosis |
| Xpert MTB/RIF assay | Rapid results, High sensitivity and specificity, Drug resistance detection | Limited detection capacity, Restricting sample type range | Reduced biosafety risks, Detection of rifampicin resistance, Suitability for multiple sample types | Active pulmonary tuberculosis and rifampicin resistance |
| Tuberculin skin test | Ease of performance, Cost-effectiveness, Rapid results | Low specificity, False negatives | Auxiliary diagnosis of tuberculosis, Evaluation of BCG vaccination effectiveness | Latent tuberculosis infection |
| Interferon gamma release assay | High specificity, Rapid results, Suitability for high-risk populations | False negatives, Short detection window,Complex interpretation | Immune response basis, Unaffected by BCG, Aiding active TB diagnosis | Latent tuberculosis infection |
