# Supplementary material for: Recent progress in tuberculosis diagnosis: insights into blood-based biomarkers and emerging technologies
Source: Front Cell Infect Microbiol. 2025 May 8;15:1567592. doi: 10.3389/fcimb.2025.1567592 (PMC12094917; doi:10.3389/fcimb.2025.1567592)
Supplement: Supplementary file 2 [file Table2.docx]

Table S2. Analysis of the Characteristics of Various emerging Technologies

| Diagnostic technology | Sensitivity | Specificity | Advantages | Limitations | Applicability |
| --- | --- | --- | --- | --- | --- |
| Automated microscope system | 85.7% | 96.9% | Reducing manual operation, Improving detection efficiency, Rapid screening | Specialized equipment requirement,Technical support dependency | Suitable for high-burden areas or resource-limited primary care |
| PET-CT Imaging | 82.6% | 67.3% | High sensitivity,Comprehensive lesion assessment | High costs, Low specificity | An auxiliary diagnostic tool |
| Xpert MTB/RIF Ultra | 76% | 95% | Rapid diagnosis,high sensitivity and specificity | High costs,High equipment and power supply requirements | Rapid diagnosis, Drug resistance testing |
| Line Probe Assay | 90.3%-100% | 95.4%-100% | High sensitivity and specificity,Rapid detection | Sputum sample dependency,Requirement for skilled technical personnel | Rapid drug resistance testing |
| Targeted Next-Generation Sequencing | 94.1% | 98.1% | Detection of multiple drug resistances,High sensitivity and specificity | High costs, High technical requirements | Detection of multiple drug resistances |
