# Supplementary material for: Recent progress in tuberculosis diagnosis: insights into blood-based biomarkers and emerging technologies
Source: Front Cell Infect Microbiol. 2025 May 8;15:1567592. doi: 10.3389/fcimb.2025.1567592 (PMC12094917; doi:10.3389/fcimb.2025.1567592)
Supplement: Supplementary file 3 [file Table3.docx]

### Table S3. Data on Biologic Treatments Before, During, and After Tuberculosis Testing

| Tuberculosis detection phase | Content | Specific measures | Precautions |
| --- | --- | --- | --- |
| Pre-TB Testing | Screening Requirements | TB exposure history, TB symptom checks | Comorbidities |
| During-TB Testing | TB Diagnosis | Skin testing/ IGRA,Xpert MTB/RIF, Sputum culture, Chest X-ray | TNF-α inhibitors, IL-17/IL-23 inhibitors |
| Post-TB Testing | Active TB | Standard Treatment Protocol | TNF-α inhibitor, Immunosuppression |
|  | LTBI | 9 months of INH or 3–4 months of RFP/RPT |  |
